# An Update Review of Approaches to Multiple Action-Based Antibacterials

**DOI:** 10.3390/antibiotics12050865

**Published:** 2023-05-06

**Authors:** John B. Bremner

**Affiliations:** School of Chemistry and Molecular Bioscience, University of Wollongong, Wollongong, NSW 2522, Australia; jbremner@uow.edu.au

**Keywords:** multiple-action design, antibacterials, combinations, hybrids, prodrugs, antibacterial resistance

## Abstract

Many approaches are being pursued to address the major global health challenge posed by the increasing resistance of pathogenic bacteria to antibacterial agents. One of the promising approaches being investigated includes the design and development of multiple action-based small-molecule antibacterials. Aspects of this broad area have been reviewed previously, and recent developments are addressed in this update review covering the literature mainly over the past three years. Considerations encompassing drug combinations, single-molecule hybrids and prodrugs are summarised in regard to the intentional design and development of multiple-action agents with a focus on potential triple or greater activities in bacteria. The hope for such single agents or combinations of single agents is that resistance development will be significantly hindered, and they may be useful in tackling bacterial disease caused by both resistant and non-resistant bacteria.

## 1. Introduction

It is now well established that the increasing resistance of pathogenic bacteria to various antibiotics poses a concerning threat to the effective treatment of bacterial disease worldwide [1,2]. Resistant bacterial strains continue to appear and spread, one example being the recent detection of a strain of *Neisseria gonorrhea* in the United States with reduced susceptibility to all the available antibiotics normally used for treatment [3], highlighting the urgent need for alternative treatments. Another recent concerning factor that has been identified is the fact that quite different drugs to antibacterials, for example widely prescribed antidepressants, can mediate increased resistance and persistence in bacteria, with resistance to many antibiotics being involved [4,5]. From phenotypic and genotypic analytical data, the heightened generation of reactive oxygen species (ROSs) after exposure to antidepressants was directly linked to the increase in resistance seen. Increased production of ROSs is then thought to activate bacterial defence mechanisms which subsequently leads to a greater ability to survive further exposure to antibiotics.

Because antimicrobial resistance is a multi-faceted and evolving global challenge, many approaches and treatment regimes are being investigated. One of the approaches involves the search for new, small-molecule antibacterials, particularly new structural entities with potentially different modes of action, with a view to counter or slow antibacterial resistance development [6,7,8,9]. Compounds with known activities in another area are also being assessed as potential new antibacterials, as exemplified by an interest in anthelmintics with a potential for countering Gram-negative bacilli-induced bacterial infections [10], rather than starting from scratch in terms of looking for or developing new hit compounds. In this context, the salicylamide family of anthelmintics such as niclosamide and nitazoxanide, which interfere with the oxidoreductase-mediated electron-transfer process in protozoa, are of interest. These compounds hold promise for antibacterial use either alone or in combination with another antibiotic, for example, colistin [10]. Possibilities for merging the repurposing developments and drug combination data with simpler antibiotics to inform future single-molecule hybrid or prodrug designs are evident here.

A sub-theme for research in the small molecule antibacterial area has been to look for multiple-action-based compounds and there has been considerable effort put into the design and development of such small molecules. Combinations of antibiotics are designed to achieve this type of outcome, but single agents that can realize multiple activities through multi-targeting may have significant advantages over combinations of two or more separate agents, especially from a pharmacokinetic point of view. This has resulted in a drive to identify potent new multiple-action-based single agents. Drug combinations, however, are very useful in informing the design process for antibacterial hybrids or hybrid prodrugs and hence are also considered in this update review. There have been a number of reviews in the area, especially of dual-action antibacterials. Other detailed reviews [11,12] and a recent book [13] have concentrated more on triple or higher action compounds or multi-target avenues and design approaches and suggestions for future synthesis. The excellent review by Gray and Wenzel [11] includes a thorough treatment of multi-target antibiotics in clinical use or with a good prospect of moving to the clinic, together with relevant combination studies. In this update review, advances in this area of ways to attain multi-targeting are summarized with an assessment of the literature in the past few years, mainly from 2021 to early 2023, and generally with a focus on medicinal chemistry aspects. It is an area that continues to grow and is likely to yield some new selective antibacterials to tackle both resistant and non-resistant bacterial pathogens in the future.

Searching the literature in this general area has difficulties as a range of terms are used including multi-targeting (multitargeting) or multi-action, polytherapy, or polypharmacology but a selection of papers is used in this update review to bring out some of the main themes and directions. The term antibacterial as well as antibiotic also need to be considered in any literature search processes, as well as antibacterial hybrids, combinations of bio-actives, and antibacterial prodrugs. New insights can be revealed (or it is advantageous, especially from a molecular design perspective) by not only considering outcomes from the compound targeting of, and interactions with, biological macromolecular targets overall but also by looking at compound–target interactions at the molecular level. Keeping both in mind can suggest possible new initiatives but radical ‘outside the square’ thinking is urgently needed. Some aspects of possible future directions are also considered in this update review.

## 2. Multi-Targeting Combinations

While combinations involve more than one compound, having such combinations does not always result in synergistic outcomes, and sometimes there is only an additive effect, or even antagonism can result in giving a poorer outcome. It is now clearly established that just mixing different antibacterials or antibacterials with non-antibacterial adjuvants may not always be advantageous and effects can be more complicated than initially thought. Often one has to consider the modes of action of the individual components first to inform the intentional choice of components in the combination in the search for synergism. A recent review by Si et al. [14] highlights this intentional approach with regard to the chemical structural design and roles of adjuvants targeting major resistance mechanisms, namely β-lactamase inhibitors, efflux pump blockers, and compounds that can permeabilize the outer membrane in bacteria. In this review, the case is also made for the simultaneous, or near simultaneous, interference with multiple resistance pathways to counter multidrug resistance [14]. Further recent developments in the use of efflux pump inhibitors in combination with antibacterials has involved the use of Gram-negative pump inhibitors, such as analogues of phenylalanine-arginine-β-naphthylamide (PaβN) which inhibit pumps in the resistance-nodulation-cell division (RND) family which can significantly potentiate the antibacterial activity of levofloxacin against *Pseudomonas aeruginosa* both in vitro and in vivo [15].

Another factor that needs to be considered and requires further study is the effect of bactericidal combinations on bacterial clearance over time particularly at concentrations which are clinically apposite [16]. This detailed study on the longer-term survival of *Staphylococcus aureus* after exposure to dual (pairwise; 14 antibiotics) as well as higher-order antibiotic combinations revealed some perhaps unexpected outcomes. For example, with certain combinations, reciprocal suppression was seen in which the efficacy of the drug combination was weaker than any of the single drugs themselves. In addition, clearance efficacy decreased as more drugs were included in the combination rather than increased, in contrast to other growth inhibitory effects and early bactericidal effects [16].

### 2.1. Recent Trends with Antibacterial Combinations

Recent trends with combinations include work on ways to counter resistance and reduce side effects seen with aminoglycosides [17]. In this review, the latest developments are summarized with respect to antibacterial combinations and non-antibacterial combinations with aminoglycosides resulting in multi-targeting and multiple effects. Other antibiotics in the combination can also be potentiated by aminoglycoside antibiotics as seen in polymyxn B with the aminoglycoside amikacin in the inhibition in vitro of polymyxin-sensitive and polymyxin-resistant *Pseudomonas aeruginosa* strains [18], or non-antibiotics can be included in the combination which also results in a reduction in toxic side effects [19]. Significant negative effects on the intermediates involved in lipopolysaccharide synthesis were seen with this combination. Aminoglycosides have also been used in combination with other non-antibiotic drugs to ameliorate toxic side effects (nephrotoxicity) from the former while still showing synergistic activity [19].

In an extension of previous work, the effectiveness of a triple combination of the antibiotics polymyxin B, trimethoprim, and rifampin has also been described against clinical isolates of ocular-sourced *Pseudomonas aeruginosa* [20]. This combination was very effective in vitro against a wide range of isolates, as a result of the different mode of action types possible, namely cell membrane disruption (polymyxin B), inhibition of DNA synthesis (trimethoprim), and interference with DNA transcription via binding to bacterial RNA polymerase (rifampin). In a different work, a triple combination of sodium ascorbate, apo-transferrin, and the antibiotic imipenem was shown to be effective in the treatment of *Acinetobacter baumannii* resistant to carbapenems in a mouse model of pneumonia mediated by this bacterium. Interestingly, a synergistic effect was demonstrated only with the triple combination rather than the dual combination possibilities in this study [21].

### 2.2. Nanoparticulates in Combinations

There is continuing interest in the use of nanoparticulate materials as components in combination with antibacterial activity. One such example is that of cubosomes, which are nano-structure particles made by a self-assembly process involving amphiphilic molecules or surfactant-like compounds. This process results in liquid crystalline phases which adopt cubic crystalline symmetry. Their structures allow for the inclusion of various types of molecules and as a result, they are of great interest with respect to drug delivery. A 2022 review by Umar et al. [22] details tumor-targeted drug delivery advances with cubosomes and includes design considerations of potential relevance to the focused delivery of antibacterial agents. Intriguingly, cubosomes themselves can also assist in bacterial killing in combination separately with an antibiotic, polymyxin B [23]. When polymyxin B was incorporated in the cubosome, the antibacterial activity was not as good compared with the combination treatment; similarly, the antibacterial activity data for polymyxin B or the cubosome alone (ineffective antibacterially, MICs > 32 μg/mL) were inferior to the combination in vitro against selected strains of the problematic Gram-negative pathogenic bacteria *Pseudomonas aeruginosa*, *Acinetobacter baumannii*, and *Klebsiella pneumoniae.* It is proposed that, in the first step, attractive electrostatic interactions between polymyxin B and lipid A in the outer membrane destabilize the membrane which is then followed by further membrane disruption on lipid interchange or exchange with the cubosome.

Silver nanoparticles and silver (I) ions are also antibacterial and are involved in interacting with many different proteins as identified in detailed studies on the proteins in *Staphylococcus aureus*, particularly disrupting glycolysis and leading to an elevation in reactive oxygen species [24]. This can then lead to the re-sensitization of methicillin-resistant *S. aureus* (MRSA) to the effects of antibiotics (for example ampicillin), when given in combination with the silver nanoparticles/Ag^+^. This is an interesting approach to overcoming antibacterial resistance with the potential for extension to other resistant pathogenic strains.

Nanoparticulate chitosan has also been used in a synergistic combination with the antimicrobial alkaloid berberine demonstrating the enhanced antibacterial activity, although still only at an MIC of 50 μg/mL against both *Staphylococcus aureus* and *Bacillus subtilis* [25]. While berberine has a number of modes of action [13], an understanding of the molecular basis of this enhancement still needs further study but it is an interesting observation. In the case of *S. aureus*, the nano-chitosan may block the efficient operation of the NorA efflux pump in some way, thus preventing the efflux of berberine from the bacterial cell.

## 3. Single-Molecule Non-Cleavable Hybrids

There has been significant activity in this area mainly in dual targeting approaches and some relevant reviews include those on the identification and development of polyfunctional drugs covering mainly antibiotics [26], different types of non-cleavable antibiotic hybrids [11,27], approaches for the realization of what have been called ‘resistance-resistant’ antibacterial strategies [28], and recently approved antibiotics by the FDA (14 out of a total of 27 that entered the market from the year 2000 to 2020) which are multi-targeting in their actions [29]. In these cases, the drugs are known to interact with more than one biological component in a pathway or with other components in different biochemical pathways in bacteria. This last review also includes detailed assessments of structure–activity relationships and of polypharmacological actions of antibiotics in the lipoglycopeptide, β-lactam, and quinolone classes. The targeting by antibiotics can involve two different macromolecular sites or two or more sites on one macromolecule. A new single-molecule hybrid design is generally based on known agents or from the results of combination testing or use. In looking at the activities to incorporate in multiple-action non-cleavable molecules, it is important to include structural features with the capability to overcome a known or likely resistance mechanism, as well as having good bactericidal or bacteriostatic activity, preferably the former. This is continuing as a major area of endeavor and is showing great promise [27].

### 3.1. Natural Products

**Figure 1:**
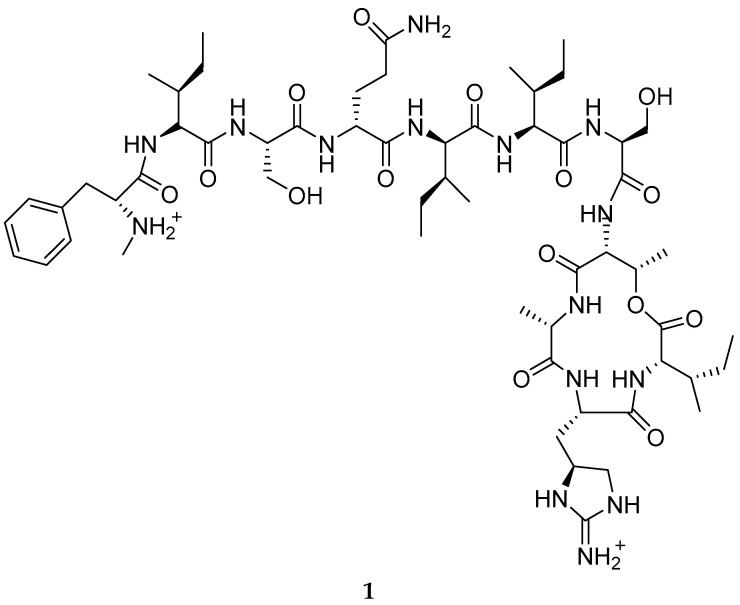
Chemical structure of the antibiotic teixobactin **1** in its diprotonated form.

Teixobactin **1** (Figure 1), a novel, large undecapeptidic antibiotic, incorporates a C-terminal thirteen-membered ring with amide and ester bonds within its structure, including a rare cationic L-*allo*-enduracididine moiety which externally presents a five-membered cyclic guanidinium group. A cationic group from *N*-methyl-D-phenylalanine is also manifest at the N-terminal. Teixobactin was isolated from the soil bacterium *Eleftheria terrae* in innovative work led by Lewis and co-workers [30] and was shown to have activity against a range of drug-resistant pathogenic Gram-positive bacteria, including methicillin-resistant *Staphylococcus aureus* and vancomycin-resistant Enterococci. It is a fascinating antibiotic whose new dual mode of action targeting the envelope of the bacterial cell has now been established in elegant work reported by Shukla et al. [31]. In their work, it was shown that supramolecular stacking or complex formation occurs subsequent to teixobactin binding to the peptidoglycan lipid II, a lipid only found in bacteria. The induced supramolecular stacks then physically displace phospholipids in the cell membrane resulting ultimately in the death of the bacterial cell. It was established that the enduracididine unit in teixobactin was responsible for the strong binding to the pyrophosphate-sugar moiety of lipid II, while at the same time, the cationic N-terminus unit interacts with a pyrophosphate group in another lipid II species. The resultant bound molecules are then favorably disposed for proceeding to the supramolecular arrangement. Such a multi-function mode of action is consistent with the fact that no bacterial resistance was seen. Supramolecular complex formation is DNA-independent which is a good resistance-avoiding strategy. It would be harder, perhaps, to develop resistance with a physical, non-enzymatic element in the mode of action as well. In terms of future studies, it may be worthwhile trying to develop simpler, lower molecular weight, non-peptide-based analogues of teixobactin, with the key interactive groups suitably disposed from the core structure. Such analogues would need to be flexible enough to obviate any possible resistance mechanisms which may develop as well, while maintaining suitable pharmacokinetic and ADMET characteristics.

**Figure 2:**
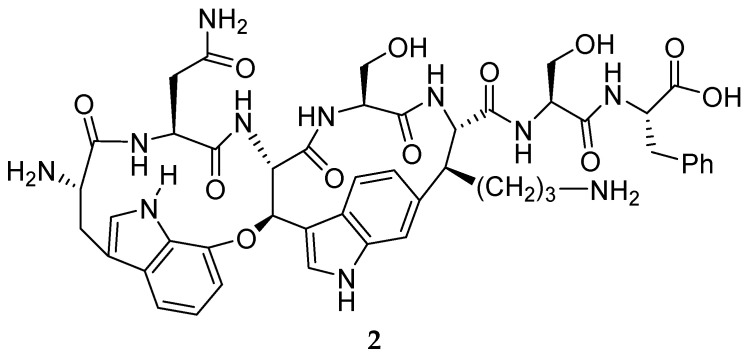
Chemical structure of darobactin A (**2**).

Another naturally occurring antibiotic identified by the Lewis group is darobactin A (**2**; Figure 2), which is produced by *Photorhabdus* bacterial symbionts in the microbiomes of entomopathogenic nematodes [32]. Darobactin A has a unique structure involving two fused large hetero-cyclophane systems, one with an embedded 3,6-indolic phane linkage and the other 3,7-indolic arrangement. This antibiotic is active against some problematic Gram-negative pathogens and acts in a new way by disrupting the cell envelope in Gram-negative bacteria through binding to the critical β–barrel outer membrane protein A (BamA), resulting ultimately in cell death [32]. The binding of darobactin D9 to BamA has now been revealed in exquisite detail through cryo-EM technology and the subsequent development of more new potent analogues [33] in which binding strength can be increased through extending opportunities for attractive interactions in the binding cleft. Darobactin D9 and a range of analogues, including some with halogenated tryptophan units, were generated by what is referred to as “biosynthetic pathway engineering” [33] using *Escherichia coli* strains.

**Figure 3:**
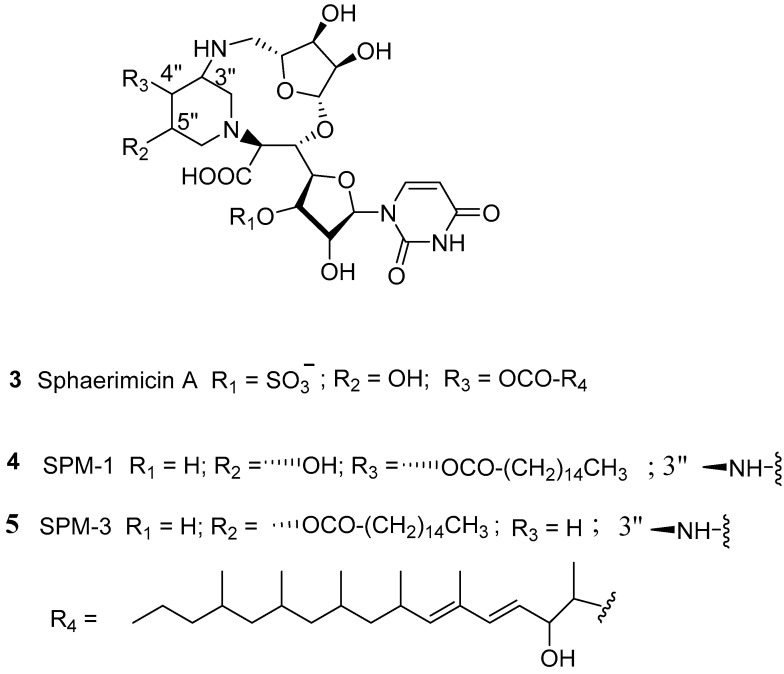
Molecular structures of sphaerimicin A (**3**) and analogues SPM-1 (**4**) and SPM-3 (**5**); stereochemistry is undetermined at some sites in **3**.

The naturally occurring, structurally complex, macrocyclic antibiotic sphaerimicin A (**3**; Figure 3) has served as the basis for the design and development of somewhat simplified analogues, which also inhibit the crucial bacterial enzyme MraY involved in cell wall construction [34]. Detailed analysis of the key binding sites to the enzyme led to the development of the analogues SPM-1 (**4**) and SPM-3 (**5**) (Figure 3). These compounds showed potent activity against Gram-positive pathogens. Because of the number of interaction sites on the enzyme, bio-mediated changes to elicit resistance are likely to be difficult. One of the sites involves a hydrophobic pocket and one suggestion for future consideration would be to change the palmityl residue to a naphthyl alkyl group for increased binding in this cleft.

The search for new antibacterial agents from unusual microbial sources is ongoing and is likely to continue in the hope of finding antibacterials whose activity has been honed through evolutionary demands [35,36] and could well involve new multi-targeting mechanisms of action.

### 3.2. Synthetic Multi-Targeting Non-Cleavable Compounds

Other advances have involved intentionally designed synthetic products usually via linking two pharmacophoric groups or molecular entities. A recent review covers part of this space looking at macrocyclic–antibiotic hybrids involving vancomycin or rifampicin linked to cephalosporin-based derivatives resulting in high-molecular-weight compounds with potent antibacterial properties through multiple actions [37]. Selected examples in this non-cleavable space are covered in this sub-section.

**Figure 4:**
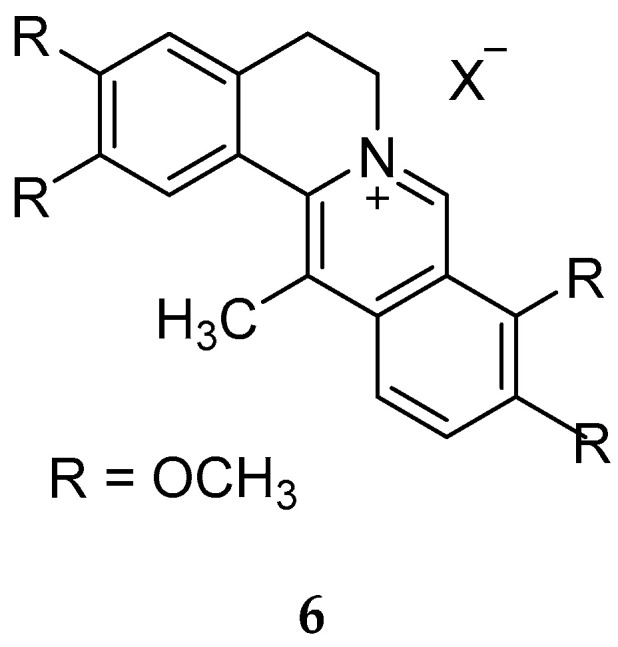
Molecular structure of the alkaloid dehydrocorydaline **6** with the counter ion X^−^ unspecified.

Conjugates of chloramphenicol-derived amine and berberine with variable-length alkyl chain linkers [38] are shown to bind to the bacterial ribosome and block protein synthesis thus mimicking the parent chloramphenicol. It was shown that one of the conjugates reduced the membrane potential in cells of *Bacillus subtilis* with the inference that the berberine component was also involved in contributing to the moderate antibacterial efficacy of this hybrid. Additionally, these hybrids did not show any significant cytotoxicity in eukaryotic cell lines. Other berberine-based hybrids have also shown encouraging activities [13], likely based on multi-action mechanisms. Interestingly, a simple protoberberine alkaloid, dehydrocorydaline **6** (Figure 4), also known as 13-methylpalmatine, is quite closely related structurally to berberine, and has potent in vivo antibacterial activity with a number of targets identified in mediating its effects on *Listeria monocytogenes* [39]. These effects include dysregulation of carbohydrate metabolism, negatively impacting cell wall synthesis as well as limiting bacterial motility [39]. This suggests a basis for hybrid design linking on a second pharmacophoric group to the core structure of **6**, perhaps through a 9-hydroxy group-based ether linkage, which might also be modulated to try and improve Gram-negative absorption. Other synthetic possibilities in the future could also include some analogues which may mimic the shape and charge distributions found in the core heterocyclic skeletons in the protoberberines and related systems through *N*-based transannular interactions. Such systems have the added possibility for different atom or group interactions with bacterial targets to improve selectivity and efficacy [40].

**Figure 5:**
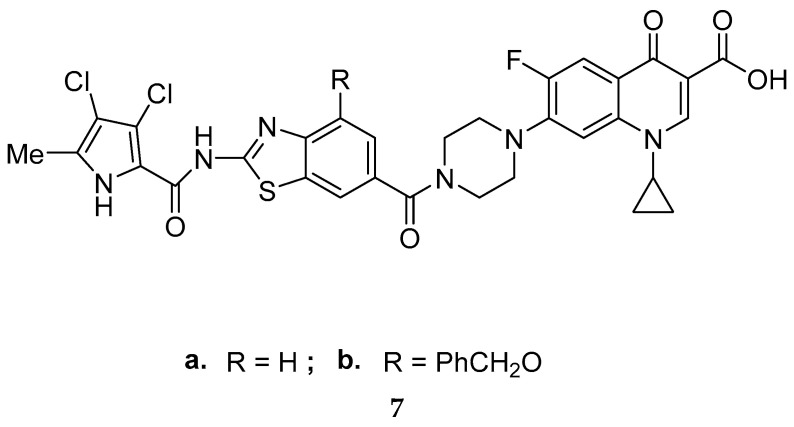
Chemical structures of the gyrase A and gyrase B inhibitor hybrids **7a** and **7b**.

A number of quite different synthetic hybrids have been reported recently based on derivatization of ciprofloxacin. Illustrative of this is the development of potent hybrid inhibitors of DNA gyrase A and B, **7a** and **7b** (Figure 5) [41]. The most potent compound revealed was **7a** against *Escherichia coli* (in vitro).

**Figure 6:**
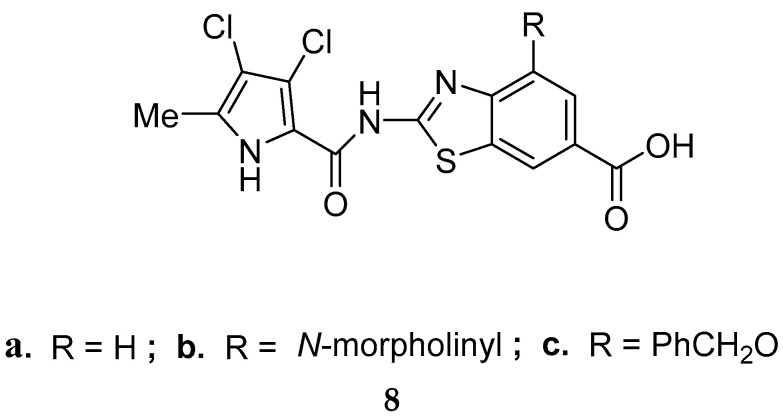
Chemical structures of the benzothiazole derivatives **8a**–**c**.

Further developments in this work resulted in more compact dual inhibitors with amide bond-linked substituted benzothiazole and pyrrole units as in **8a**–**c** (Figure 6) [42,43]. However, these compounds **8a**–**c**, which do not have a ciprofloxacin moiety, are based on two of the carboxylic acid precursors [42] used in the synthesis of **7a** and **7b**; the synthesis of the other substituted carboxylic acid **8b** is described in a subsequent paper [42]. Compounds **8a**–**c** target bacterial DNA gyrase and topoisomerase IV and have impressive broad-spectrum potency in vitro and in vivo against Gram-positive and some Gram-negative pathogenic bacteria [42,43]. The dual inhibitor **8b** had particularly favorable physicochemical properties, was selective for bacterial topoisomerases, and did not have toxicity issues. It is thus a promising candidate for further development.

**Figure 7:**
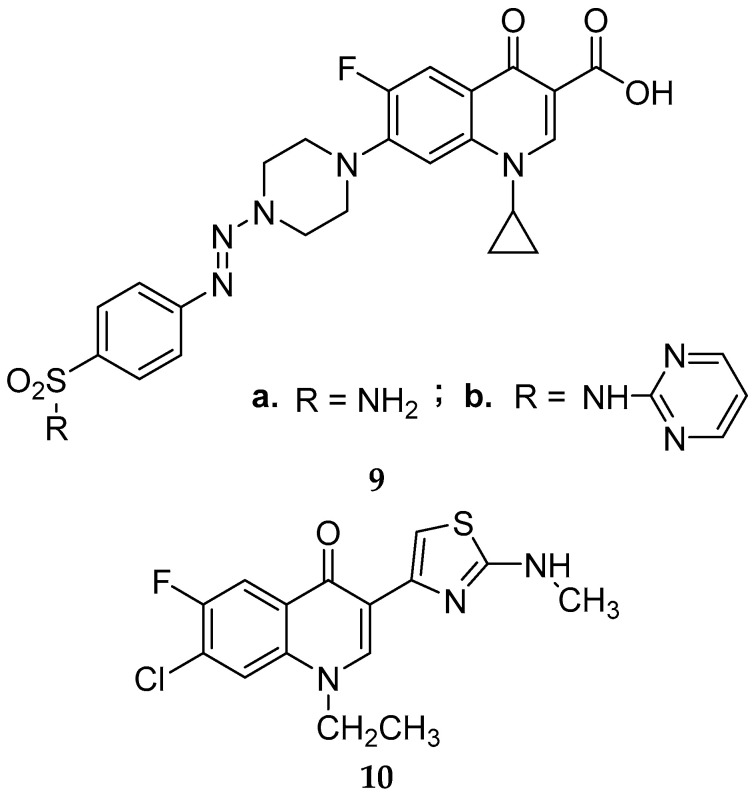
Molecular structures of quinolone-based compounds **9a**,**b** and **10**.

Further hybrids involving derivatization of ciprofloxacin at the terminal piperidine unit **9a**,**b** (Figure 7) [44] or replacement of the carboxylic acid group at the 3-position in the quinolone core as in compound **10** (Figure 7) [45] have also shown interesting antibacterial properties and modes of action, interacting with both DNA gyrase and topoisomerase IV. Compounds **9a**,**b** incorporate a terminal sulfonamide unit linked via a triazene moiety to ciprofloxacin; other compounds in this series were linked via an amide moiety and generally showed a little better in vitro activity [44]. The interest structurally with compound **10** revolves around the effective replacement of the carboxylic acid group previously thought to be essential for good antibacterial activity [45]. Interestingly, compound **10** displayed a number of actions including the inhibition of biofilm formation and negative impacts on bacterial cell membranes as well as DNA replication.

**Figure 8:**
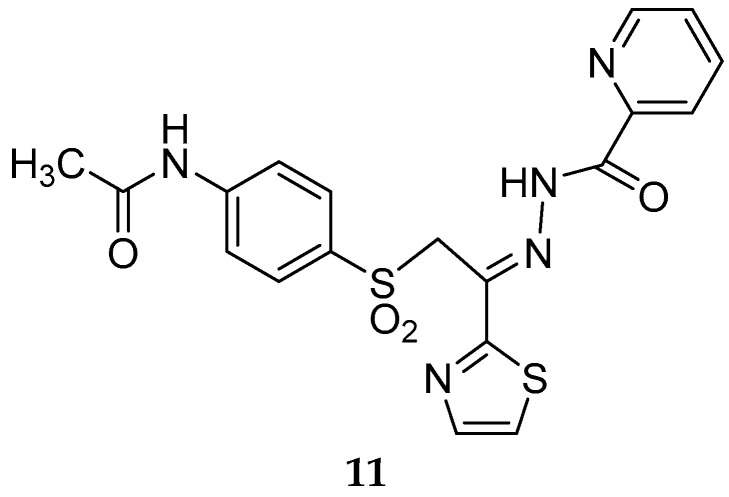
Chemical structure of the thiazolimine derivative **11**.

There is not as much work reported on intentionally triple- or greatertargeting of different sites but it is inherently a difficult area in which to achieve success. Some recent examples though include some reported in the review by Wetzel et al. [29] which mentions FDA-approved antibiotics with four activities. Quite low-molecular-weight multi-targeting agents have also been developed for the inhibition of *Enterococcus faecalis* [46]. These compounds are based on sulfathiazole, a short-acting sulfonamide antibacterial of limited current clinical application in combination with other sulfonamides. Structural modification studies around the nitrogen of the sulfonamide moiety and other substitutions have led to a new group of benzenesulfonyl thiazolimines, for example, **11** (Figure 8), with more favorable physicochemical properties (particularly water solubility) and a range of bacterial target interactions, including antibiofilm activity and membrane damage amongst others [46].

Quite different multi-targeting hybrids have been reported with substituted coumarins containing aminophosphonate groups [47] and one of the targets is bacterial DNA, with these compounds being capable of DNA-base pair intercalation. Negative impacts on the integrity of the cell membrane was another effect seen. DNA and RNA interactions are good targets for antibacterial agents if entry to the cell can be achieved. The Suckling group in the University of Strathclyde have been developing small molecule anti-infective agents which are multi-targeting in an effort to reduce resistance development. In particular, they have developed a range of nucleic acid minor groove binders which have potent and broad-spectrum activity against a range of pathogenic bacteria. This work has been reviewed in detail recently [48]. The design of these binders was based on the naturally occurring compounds distamycin and netropsin and then developed further. One such derivative developed, MGP-BP-3, showed good activity against *Staphylococcus aureus* and *Clostridium difficile* and asserts its activity through binding to the *dna*D and *mra*Y promoter regions thus inhibiting the transcription process initiated by these genes. In addition, no resistance development to MGP-BP-3 was seen in vitro with *Staphylococcus aureus.*

**Figure 9:**
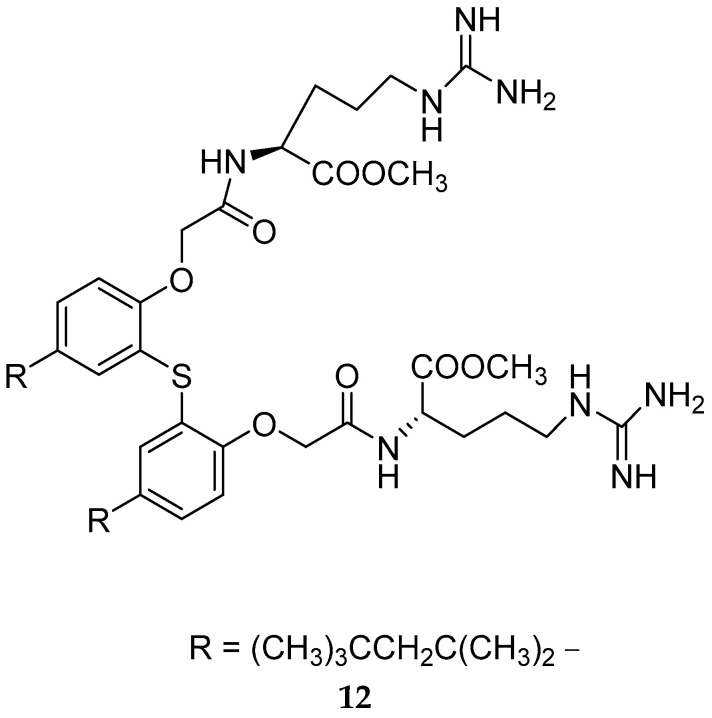
Molecular structure of the membrane targeting agent **12**.

Considerable interest has been shown in the design, synthesis and evaluation of small-molecule mimetics of antimicrobial peptides (AMPs) as potential antibacterials to overcome resistant strains because of their primary mode of action through the disruption of cell membranes. Some AMPs, however, can move across the membrane and into the cell where further interactions with proteins, DNA, or RNA can occur. AMP mimetics thus have the potential for multiple actions which might further reduce resistance development risks [49,50]. A range of mimetics have been investigated and a recent illustrative example is that of the diphenyl thioether-based derivative **12** (Figure 9) which displayed potent activity against Gram-positive bacteria in vitro, and against *Staphylococcus aureus* in a mouse in vivo model [51]. This derivative was designed with a twofold purpose in mind; one was to enhance electrostatic interactions with the bacterial cell membrane (negatively charged) via the positively charged guanidino groups (after protonation at physiological pH of these very basic groups) on the amphiphile, and the other was to include hydrophobic groups (C8 branched alkyl substituents on two aromatic rings) for interaction with, and insertion into, the phospholipid bilayer of the membrane. Negative cidal impacts on the coherence of the cell membrane would then be expected to follow these interactions.

A new commercial co-polymeric material designated RECCE^®^ 327 (R 327) and developed by Recce Pharmaceuticals Ltd. in Australia seems to be a very promising antibacterial with ‘universal’ targeting and multiple mechanisms of action [52]. It is a water-soluble material and is bactericidal to both Gram-positive and Gram-negative bacteria. The primary mode of action is through the rapid negative and non-reversible impact on energetics in the cell on stopping ATP production. Other effects include those on bacterial cell division, and permeabilization of the cell membrane (in *Escherichia coli*) [52].

A further very promising potential area for the development of multi-targeting compounds is that of metal complexes. The structural diversity of these compounds and the potential for countering antimicrobial resistance is highlighted in a recent detailed review [53]. In this review, modes of action are considered and the scope for future work is outlined. In this context, the ruthenium polypyridine complex Ru-1, which incorporates an anthraquinone group in one of the bipyridyl ligands, shows excellent in vitro activity against *Staphylococcus aureus*, comparable with vancomycin [54]. One potential advantage of metal complexes is the range of geometrical dispositions that can be achieved for pharmacophoric groups in the ligands complexed to the transition metal ion. Selective multi-targeting in a compact structural environment is an attractive design possibility. In addition, photosensitizing capabilities [53] for the subsequent generation of reactive oxygen species may enhance broad-spectrum antibacterial potencies. Metal complexes thus offer excellent potential to interact with more complex and effective binding modes to one or more bacterial target sites and thus be more active than the free ligands themselves.

Designing new potential single molecule non-cleavable agents which intentionally have a number of biological targets or interact at two distinct sites on one or more targets continues to be a challenging area. As well as the target interactions themselves, one has to consider a range of other issues among which are scalable efficient syntheses, physicochemical properties, drug administration routes, site selectivity in the body, ADMET properties, resistant bacterial strains, cell wall barriers, biofilms, persistors, and sporulation problems. Higher molecular weights in linked hybrids are also potential issues but reducing molecular weights by having a direct attachment between units with two or more pharmacophoric entities or proceeding to a partial fusion of the core structural units, offers a possible pathway to reducing molecular weights while maintaining antibacterial or other activity.

## 4. Prodrug-Based Approaches

Targeting prodrugs to bacteria or preferential uptake into bacteria, then release at or near the bacterial biological target site, affords opportunities for multiple action outcomes. An earlier thorough review of antibacterial prodrugs, which were directed towards countering bacterial resistance, was published in 2020 [55] but there has been considerable further work done from around that time. Some examples are noted in the following sub-sections.

### 4.1. Enzyme-Mediated Triggering

**Scheme 1:**
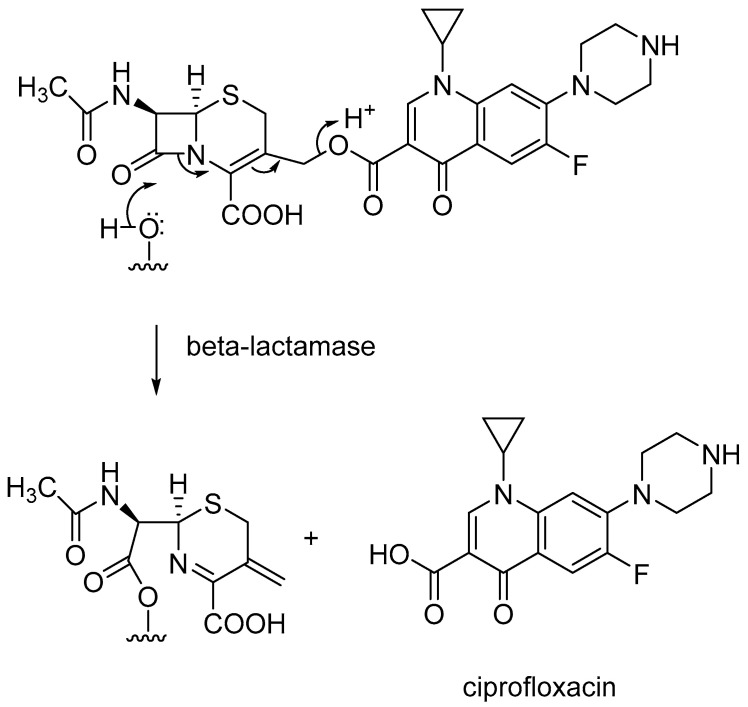
Fragmentation process to generate the antibacterial, ciprofloxacin, from a cephalosporin-based prodrug.

The common use of bacterially specific enzymes such as β-lactamases for prodrug activation has been expanded in recent years as exemplified by the work of Evans et al. [56]. These scientists have reported the design and synthesis of a novel ester-linked prodrug combining a cephalosporin (via an iodomethyl substituent group) with a fluoroquinolone antibiotic (ciprofloxacin; via the 3-carboxylic acid group). Ciprofloxacin itself, which targets both DNA gyrase and topoisomerase IV, was then released (Scheme 1) after β-lactam ring opening in the cephalosporin by β–lactamase and good antibacterial activity was seen.

Penicillin-binding proteins (PBPs) can also be involved in site-specific triggering as illustrated in the elegant work of Kelso and co-workers [57] on cephalosporin-3′-diazeniumdiolates which release nitric oxide through an elimination reaction after β-lactam ring cleavage. These diazeniumdiolates display dual antibacterial and antibiofilm activities in vitro against clinical isolates of *Pseudomonas aeruginosa.* One compound in the series was also tested in a mouse respiratory infection model with *P. aeruginosa* and showed excellent activity.

Other triggering approaches include the utilization of specific hydrolytic bacterial enzymes such as proteases in *Pseudomonas aeruginosa* [58]. The prodrug in this case includes a carbohydrate-based lectin probe head group to target *P. aeruginosa* and a tetrapeptide linker cleavable by the proteases to then release the fluoroquinolone antibiotic. These conjugates were stable in the host blood plasma and to liver metabolism but released the antibiotic in a timely manner when exposed to *P. aeruginosa*. This is a very promising antibacterial prodrug approach, although the antibiotic released will need to be one that is able to override any bacterial resistance. Resistance may develop if the specific proteases mutate so that linker hydrolysis does not proceed, but this may be less likely.

**Figure 10:**
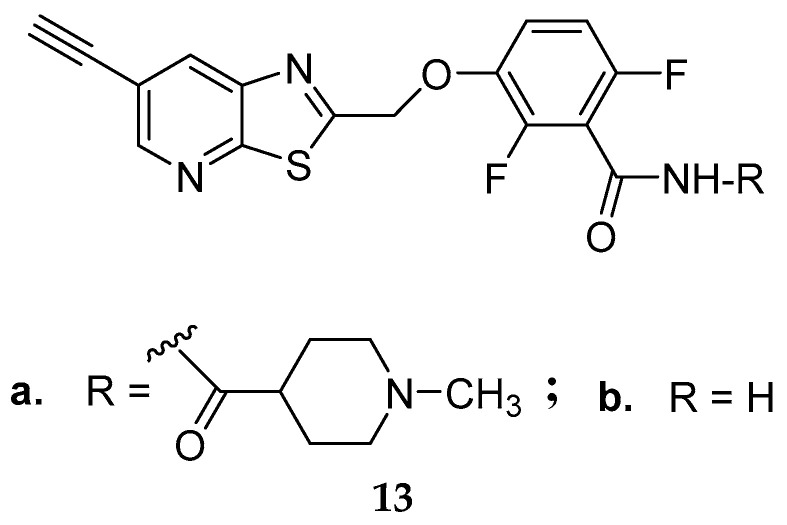
Molecular structures of the FtsZ inhibitor **13b** and its prodrug **13a**.

Carboxy ester prodrugs of phosphonate antibiotics can be specifically activated by particular esterases, in this case by GloB and FrmB in *Staphylococcus aureus* [59]. In other work, mouse serum acetylcholinesterases have been shown to rapidly release the active antibacterial **13b** (Figure 10) (TXH9179) from the imide prodrug TXH1033 (compound **13a**, Figure 10); significantly, **13a** in turn did not exhibit any mammalian toxicity in the successful treatment of an MRSA infection in a mouse model [60]. The imide moiety was used in the prodrug to improve properties for formulation and in vivo administration [60]. The active compound **13b** is a new benzamide FtsZ protein inhibitor which has been shown by X-ray crystallography to make a number of key interactions, including one involving the ethynyl substituent group, to improve binding with this vital protein in bacterial cell division.

A somewhat complicated but effective technology was recently announced aimed at the treatment of bacterial wound infections. The technology is centered on a fabricated adenosine triphosphate (ATP)-activated prodrug system, abbreviated as HiZP [61] which incorporates a zeolite framework and a polyacrylamide hydrogel microsphere which can transport indole-3-acetic acid and an oxidizing enzyme, horseradish peroxidase, in a single carrier unit without the indole being affected. The system is then activated by ATP secreted from bacteria to produce reactive oxygen species which can attack bacterial targets resulting in broad-spectrum antibacterial activity. This treatment process was shown to be effective in wound healing with virtually no side effects in a murine infection model [61].

### 4.2. Bio-Orthogonal Activation Approaches

New developments have been reported [62] in the triggering of prodrug activation, for example by using a bio-orthogonal chemistry approach. Such an approach does not rely on an endogenous enzyme for activation, as is normally done, but rather on what might be considered as enzyme-like polymer materials with incorporated transition metal catalysts referred to as polyzymes. These polyzymes can infiltrate biofilms and then activate the prodrug to release the active antibiotic within the biofilm. Alternatively, a thermo-responsive gold-based nanoparticulate system, known as a nanozyme, can be used, in which temperatures greater than 37 °C will enable a free catalyst release followed by prodrug activation [62]. These systems thus activate antibiotics with the promise of reduced side effects.

Similarly bio-orthogonal in nature is the targeted allyl ester prodrug activation by a ruthenium complex catalyst after siderophore-mediated selective uptake into the bacterial cell (*Escherichia coli*) [63]. This catalyst was then shown to be able to catalyze a prodrug allyl ester group conversion to a carboxylic acid group resulting in the release of the potent broad-spectrum antibacterial, moxifloxacin [63]. This was the first time such a prodrug activation process had been demonstrated and the approach holds considerable promise for future applications.

There have also been a number of developments in applying external bio-orthogonal triggers, such as light, for activation. For the light-based process in photodynamic therapy, photosensitizers are required, and the non-porphyrinoid ones have been comprehensively reviewed [64]. With photosensitization, a cascade effect comes into play after light exposure with ground-state triplet oxygen being excited to the highly reactive singlet oxygen after energy transfer from the excited photosensitizer. Singlet oxygen and other derived reactive oxygen species can then attack a number of bacterial targets. Selectivity depends on the uptake into the bacterial cell as well as control over light exposure. Photosensitizer in-cell concentrations can be compromised by the activity of bacterial efflux pumps but a possible solution to this has been reported, involving attachment of an efflux pump blocking moiety to the photosensitizer in the case of methylene blue [65,66].

### 4.3. Release of Sulfur Dioxide

In the important area of gaseous antibacterials, another interesting, targeted prodrug has been reported in which a siderophore has been linked to a moiety capable of releasing sulfur dioxide on nucleophilic attack by a thiol group as in glutathione [67]. The two-photon light-induced release of sulfur dioxide can also result from *o*-nitrophenyl alkyl-substituted derivatives [68,69]. Synchronous release of sulfur dioxide, which can inhibit ATP synthesis in the cell amongst other detrimental actions, together with another broad-spectrum antibacterial, ethyl ferulate, resulted in very good antibacterial activity against *Enterobacter cloacae* [69]. Interestingly, a synergistic antibacterial response was seen based on a significantly lower MIC after irradiation, compared with that prior to light exposure and with the MIC of ethyl ferulate itself. It is also worth noting though that a third compound, 2-nitroso-4,5-dimethoxybenzaldehyde, would also be formed from the cleavage process and it may possibly also have antibacterial effects, although the simpler analogous compound, 2-nitrosobenzaldehyde, was not active against *Escherichia coli* K12 in vitro [70]. In the future, adverse effects on host tissues in vivo will need to be assessed as well for the released compounds, as it is possible that they could leak out and be distributed more widely.

### 4.4. Future Antibacterial Prodrug Developments

**Scheme 2:**
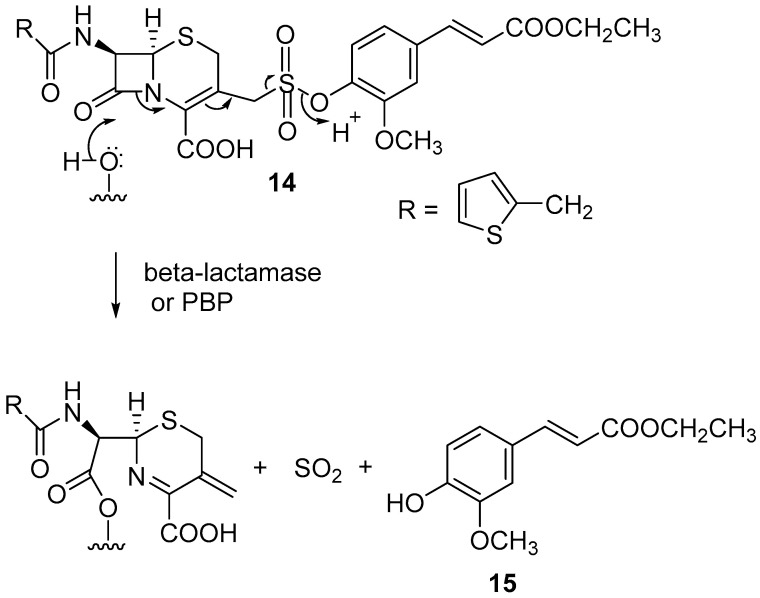
Suggested prodrug fragmentation process to generate two antibacterial compounds.

There is much more scope for new developments with prodrug approaches. For example, prodrugs that target bacteria and then can be triggered to release two or more active components within the bacterial cell or in the cell surface region, particularly if one or more of the released components has multiple actions. This should then be very difficult to counter from a resistance perspective and be less likely to have a negative effect on host cells. In this context, further attention might usefully be given to having a gas such as sulfur dioxide as one of the released components (see Section 4.3). Antibacterial gas therapy is the subject of a recent review which includes sulfur dioxide generation from prodrugs [68]. One possibility here that may be worth pursuing is to assemble a prodrug (for example **14**, Scheme 2) from a cephalosporin-based head group to which is attached a group capable of fragmenting via enamine-initiated elimination to produce SO_2_ in situ after β-lactamase (or possibly PBP binding)-induced β-lactam ring opening, plus a second antibacterial agent capable of interacting synergistically with other bacterial target sites. This is illustrated in Scheme 2 with ferulic acid ethyl ester (ethyl ferulate, **15**) as the phenolic antibacterial agent tail group [69], but a range of other phenolic or hydroxyl group-containing antibacterials, or suitably substituted derivatives to facilitate cell penetration, could be considered here. Similarly, the cephalosporin component could be varied with different R groups. Such a prodrug fragmentation should be a bacterially specific and effective process and hopefully would obviate some of the challenges with light-induced activation processes.

## 5. Conclusions

There is much scope for selective protein destruction strategies mediated by bacterial PROTACS (BacProtacs) as discussed in a recent review of the area by Venkatesan et al. [71] and a preview by Burslem [72], in which relatively small heterobifunctional molecules are highlighted as promising BacPROTACS. Morreale et al. [73] have developed the BacProtac technology with small molecules and this approach should be very useful in informing the development of powerful new antibiotics or of combinations with different antibacterial effects.

There is also much scope in the search for new antibiotics from natural sources potentially with multi-targeting capabilities [35]. One aspect of this search might be to look further at whether some bacteria produce prodrug-type compounds which are only activated on encountering another competitor. Such compounds could provide new avenues for synthetic antibacterial prodrug designs.

In the synthetic area, out-of-the-box thinking has resulted in the proof of principle development of intriguing light-activated mini-molecular machines which disrupt bacterial cell membranes [74]. It is considered likely that bacterial resistance development will be very slow with such machines.

Finally, the application of Artificial Intelligence (AI)-based technologies is an emerging area in drug design, and it is likely to see further applications in the design and synthesis of new multiple-action-based antibacterials or prodrugs. One wonders if AI systems can also be trained to ‘think outside the box’ in a non-human way and suggest new structures beyond those on which they may be trained and suggest implementation pathways. This is a fascinating and significant area of research for the future.
